# Transcription elongation regulator 1 (TCERG1) regulates competent RNA polymerase II-mediated elongation of HIV-1 transcription and facilitates efficient viral replication

**DOI:** 10.1186/1742-4690-10-124

**Published:** 2013-10-28

**Authors:** Mayte Coiras, Marta Montes, Immaculada Montanuy, María Rosa López-Huertas, Elena Mateos, Caroline Le Sommer, Mariano A Garcia-Blanco, Cristina Hernández-Munain, José Alcamí, Carlos Suñé

**Affiliations:** 1 AIDS Immunopathology Unit, Centro Nacional de Microbiología, Instituto de Salud Carlos III, Majadahonda, Madrid, Spain; 2 Department of Molecular Biology, Instituto de Parasitología y Biomedicina “López Neyra” (IPBLN-CSIC), Armilla, Granada 18016, Spain; 3 Department of Molecular Genetics and Microbiology, and Center for RNA Biology, Duke University Medical Center, 213 Research Drive, Durham, NC 27710, USA; 4 Department of Cell Biology and Immunology, Instituto de Parasitología y Biomedicina “López Neyra” (IPBLN-CSIC), Armilla Granada 18016, Spain; 5 Biotech Research and Innovation Centre, University of Copenhagen, Ole Maaløes Vej 5, Copenhagen DK-2200, Denmark; 6 Department of Medicine, University of Cambridge, Hills Road, Cambridge CB2 2QQ, United Kingdom; 7 The Hamner Institutes for Health Sciences, 6 Davis Drive, Research Triangle Park, Durham, NC 27709, USA

**Keywords:** TCERG1, Transcription elongation, RNA polymerase II, Pausing

## Abstract

**Background:**

Control of RNA polymerase II (RNAPII) release from pausing has been proposed as a checkpoint mechanism to ensure optimal RNAPII activity, especially in large, highly regulated genes. HIV-1 gene expression is highly regulated at the level of elongation, which includes transcriptional pausing that is mediated by both viral and cellular factors. Here, we present evidence for a specific role of the elongation-related factor TCERG1 in regulating the extent of HIV-1 elongation and viral replication *in vivo*.

**Results:**

We show that TCERG1 depletion diminishes the basal and viral Tat-activated transcription from the HIV-1 LTR. In support of a role for an elongation mechanism in the transcriptional control of HIV-1, we found that TCERG1 modifies the levels of pre-mRNAs generated at distal regions of HIV-1. Most importantly, TCERG1 directly affects the elongation rate of RNAPII transcription *in vivo*. Furthermore, our data demonstrate that TCERG1 regulates HIV-1 transcription by increasing the rate of RNAPII elongation through the phosphorylation of serine 2 within the carboxyl-terminal domain (CTD) of RNAPII and suggest a mechanism for the involvement of TCERG1 in relieving pausing. Finally, we show that TCERG1 is required for HIV-1 replication.

**Conclusions:**

Our study reveals that TCERG1 regulates HIV-1 transcriptional elongation by increasing the elongation rate of RNAPII and phosphorylation of Ser 2 within the CTD. Based on our data, we propose a general mechanism for TCERG1 acting on genes that are regulated at the level of elongation by increasing the rate of RNAPII transcription through the phosphorylation of Ser2. In the case of HIV-1, our evidence provides the basis for further investigation of TCERG1 as a potential therapeutic target for the inhibition of HIV-1 replication

## Background

Gene transcription begins with the recruitment and assembly of an RNA polymerase II (RNAPII) complex on core promoter sequences, followed by initiation of RNA synthesis and subsequent elongation. Most of the initial studies to improve the understanding of transcriptional regulation were focused on the initiation stage and the mechanism by which the RNAPII complexes were recruited and assembled on promoters (reviewed in [1]). However, transcription elongation is also a major target for gene regulation. Initial studies of the *Hsp70* gene in Drosophila have shown that RNAPII complexes stall in the 5′ region of the transcription unit [2]. Paused RNAPII within the promoter-proximal region has also been observed at many proto-oncogenes, such as *c-myb*[3], *c-mos*[4], *c-myc*[5], and *c-fos*[6], and other mammalian genes [7-9], suggesting that RNAPII pausing at promoter-proximal regions is predominantly the rule rather than the exception. More recently, genome-wide results from humans and Drosophila have provided strong evidence for the widespread existence of paused RNAPII at gene promoter-proximal regions [10-15]. Thus, increasing evidence suggests that RNAPII often arrests within the vicinities of the start site of transcription and that release of paused RNAPII might act as an important checkpoint to ensure optimal RNAPII activity, especially in large, highly regulated genes (reviewed in [16]). In addition to promoter-proximal pausing, two recent reports using global analysis of nascent RNA in yeast have shown that RNAPII transiently accumulates at specific sites before termination and around 3′ splice sites of genes [17,18]. Although the nature and position of the observed pausing differ between the studies, a strong correlation between paused RNAPII and co-transcriptional splicing was observed. These observations agree with the model in which transcriptional pausing acts as a regulatory checkpoint, in this case for a downstream processing event.

Differences in the phosphorylation status of the tandem repeats of the consensus heptapeptide YSPTSPS in the carboxyl-terminal domain (CTD) of the largest subunit of RNAPII have been associated with the location of RNAPII on the gene. Hypophosphorylated RNAPII assembles into pre-initiation complexes at gene promoters, whereas hyperphosphorylated RNAPII associates with the elongation stage. Cyclin-dependent kinase 7 (CDK7), which is part of the general transcription factor IIH (TFIIH), is responsible for the phosphorylation of serines at the fifth position (Ser5). This modification has been linked with transcription initiation and is preferentially associated with the 5′-end of the genes. As RNAPII elongates further downstream, phosphorylation of serines at the second position (Ser2) increases, which is viewed as an elongation mark and is preferentially associated with the 3′-end of the genes. The transition between Ser5 and Ser2 phosphorylation is essential for the release of paused RNAPII complexes from the proximal promoter and represents an important mechanism of control for proper elongation. Extensive phosphorylation of Ser5 has also been found in the CTD of RNAPII paused around 3′ splice sites of genes [17]. Phosphorylation of Ser2 of the CTD could conceivably also be associated with release from this pausing. Although the correlation between Ser2 phosphorylation and elongation is widely accepted, recent discoveries related to modifications of the CTD and the enzymes involved show that this model is an oversimplified representation of a more complex process regulating elongation control of gene expression (reviewed in [19-21]).

Human Immunodeficiency virus type 1 (HIV-1), the etiologic agent of acquired immunodeficiency syndrome (AIDS), is a complex lentivirus with a highly regulated life cycle. Upon infection, HIV-1 integrates into the host genome DNA where it remains in a silent state, which is critical in latently infected cells. The chromatin structure, as well as a specific group of proteins, plays a role in repressing viral gene expression by causing the RNAPII complexes to pause in the 5′ region of the transcription unit. It is now well established that Nuc-1, a nucleosome located immediately downstream of the HIV-1 transcriptional initiation site, directly represses promoter activity and is mainly responsible for transcriptional silencing in latently infected cells. Consequently, the synthesized transcripts fail to support viral replication [22]. In recent work, a mechanism involving the microprocessor complex as well as specific termination factors has been proposed to regulate pausing and premature termination at the HIV-1 Long Terminal Repeat (LTR) [23]. HIV-1 transcription is controlled by *cis*-acting sequences located at the 5′ LTR and by both viral and cellular *trans*-acting factors [24]. Among other sequences, the LTR contains a canonical TATA-box sequence followed by two NF-κΒ and three Sp1 sites that constitute the core promoter and modulate basal and activated viral transcription [25]. Activation of this promoter requires expression of the viral regulatory protein Tat, which binds to a 59-nt stem-bulge-loop structured RNA element named TAR (for *trans*-activation-responsive region) located at the 5′-end of all HIV-1 nascent transcripts [26]. The transcriptional *trans*-activation of the HIV-1 promoter by Tat requires specific cellular cofactors. The complex P-TEFb is a Tat cofactor that is essential for the transcriptional function of Tat [27-29] and is recruited to the TAR element through the interaction of Tat with cyclin T1 (a component of P-TEFb). The CDK9 component of P-TEFb phosphorylates Ser2 within the CTD of RNAPII as well as several positive and negative elongation factors. These events lead to release of the arrested RNA complexes in the 5′ region of the transcription unit, resulting in a potent stimulation of the overall rate of transcriptional elongation and productive HIV-1 replication. In addition to the essential role of the Tat/TAR axis in the control of HIV-1 gene expression and latency, the HIV TATA-box and immediately flanking sequences have been shown to be specifically required for the production of specific pre-initiation complexes that are targets of *trans*-activation by Tat [30-35]. In this context, Tat plays a highly specific role in the recruitment of transcription factors and co-activators through its multifaceted interactions with different cellular components [36-44]. Therefore, HIV-1 transcription is a critical step in the viral life cycle, and the intricate regulation of this transcription provides the basis for understanding viral transcriptional latency [45-48].

In addition to P-TEFb, other factors have been implicated in the mechanism of Tat-mediated transcriptional activation. Among these, TCERG1 (HUGO approved gene name: transcription elongation regulator 1; previously designated CA150, for co-activator of 150 kDa) is a nuclear protein that was first detected in a fraction from a wild-type Tat affinity column that contained an activity required for transcription activation by Tat in an *in vitro* transcription system [49]. We have previously shown that immunodepletion of TCERG1 from nuclear extracts decreases Tat activation of RNAPII elongation efficiency *in vitro*, suggesting that TCERG1 might play a positive role in this process [49]. We have also shown that transient overexpression of TCERG1 protein in HEK293T cells reduces the activity of HIV-1 basal and Tat-activated transcription of the HIV-1 LTR by decreasing the level of elongation-competent transcription complexes. We hypothesized that overexpression of TCERG1 might alter the composition of the Tat-responsive transcription complexes, e.g. by sequestering a limiting Tat-cofactor [50]. Based on these and other data, such as the interaction of TCERG1 with several components of the elongation machinery [51] and with the phosphorylated CTD of RNAPII [52], TCERG1 appears to function in the elongation stage of transcription. The aforementioned data strongly suggested that TCERG1 is a cofactor for HIV-1 transcriptional elongation and thus might be a relevant protein in the HIV-1 life cycle. However, this hypothesis has not previously been formally tested *in vivo*.

Very recently, we have demonstrated that TCERG1 regulates the expression of the apoptosis gene *Bcl-x* by modulating the rate of RNAPII transcription. In this previous work, we proposed that TCERG1 acts on RNAPII to relieve pausing, thus acting as a checkpoint factor to ensure optimal RNAPII activity during elongation [53]. These results posit the question of whether the effect of TCERG1 on *Bcl-x* elongation is a more general mechanism that operates in other genes that harbor pause sites, e.g., in HIV-1. In this manuscript, we evaluate the role of endogenous TCERG1 in HIV-1 transcription and viral replication *in vivo*. We found that TCERG1 depletion diminishes the basal and Tat-activated transcription from the HIV-1 LTR by impairing elongation of the HIV-1 transcripts. Most importantly, TCERG1 affects the rate of RNAPII transcription of HIV-1. We also observed that the TCERG1-mediated transcriptional effect is associated with changes in the pattern of Ser2 phosphorylation in the RNAPII CTD. In addition, we show that TCERG1 is required for HIV-1 replication, as depletion of TCERG1 decreased viral replication in Jurkat cells and peripheral blood lymphocytes (PBLs). Based on our data, we propose a general mechanism for TCERG1 acting on genes that are regulated at the level of elongation by increasing the rate of RNAPII transcription through the phosphorylation of Ser2. In the case of HIV-1, our evidence provides the basis for further investigation of TCERG1 as a potential therapeutic target for the inhibition of HIV-1 replication.

## Results

### TCERG1 depletion decreases basal and Tat-activated transcription from the HIV-1 LTR

**Figure 1:**
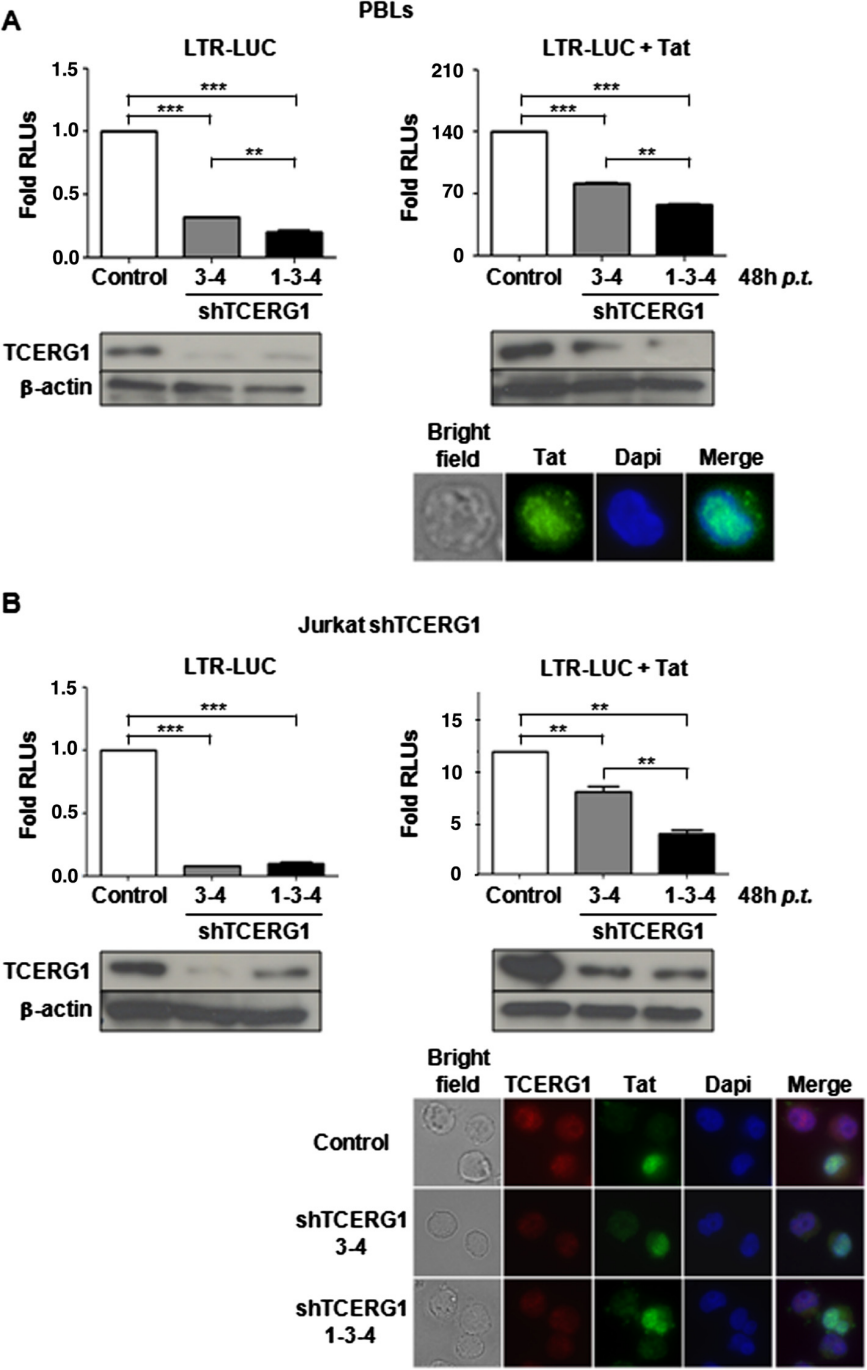
**TCERG1 depletion decreases basal and Tat-activated transcription from the HIV-1 LTR. A.** PBLs were co-transfected with a luciferase expression vector under the control of the HIV-1 LTR promoter (LTR-LUC) along with pGeneClip-shTCERG1-C1 or pGeneClip-shTCERG1-3 and pGeneClip-shTCERG1-4, and the latter pair together with pGeneClip-shTCERG1-1. The luciferase activity was quantified 72 hours after transfection as relative light units (RLUs) using the control cells as the basal signal. Data from three independent experiments are represented in the histogram (means ± SEM). Statistical analysis was performed and *p*-values are shown (**, *p <* 0.01; ***, *p <* 0.001). TCERG1 interference was determined by immunoblotting. Appropriate expression and nuclear localization of Tat was evaluated by immunofluorescence using a specific antibody against Tat and Dapi to stain the nucleus (a representative cell is shown). **B.** Jurkat cells with stable interference of TCERG1 were co-transfected with a luciferase expression vector under the control of the HIV-1 LTR promoter (LTR-LUC) along with CMV-Tat101 or pcDNA3 as negative control. The luciferase activity was quantified 72 hours after transfection as relative light units (RLUs) using the control cells as the basal signal. The results of three different experiments are represented in the histogram (means ± SEM). Statistical analysis was performed, and *p*-values are shown (**, *p <* 0.01; ***, *p <* 0.001). TCERG1 interference was determined by immunoblotting. The effect of TCERG1 interference on the expression and nuclear localization of Tat was analyzed by immunofluorescence in all Jurkat cell lines with stable interference of TCERG1.

As a first approach to test the role of TCERG1 in HIV-1 transcription, we examined PBLs co-transfected with a luciferase expression vector under the control of the HIV-1 LTR promoter along with the following vectors to induce TCERG1 mRNA interference: pGeneClip-shTCERG1-C1 or pGeneClip-shTCERG1-3 and pGeneClip-shTCERG1-4, and the latter pair together with pGeneClip-shTCERG1-1. TCERG1 knockdown was assessed by quantitative RT-PCR assay (qRT-PCR) (Figure 1A). PBLs were transfected in a resting state and then maintained with IL-2 for 48 hours. Upon quantification of the luciferase activity, we observed that TCERG1 knockdown significantly reduced the basal transcription of the LTR (Figure 1A, left panel). Similar results were observed when the experiment was performed in the presence of Tat. Tat-mediated transcriptional activation of the luciferase reporter gene was reduced upon TCERG1 depletion in PBLs (Figure 1A, right panel). Tat expression in the nucleus of the transfected cells was assessed by immunofluorescence (Figure 1A).

To ensure long-term, reproducible, and defined silencing effects, we generated Jurkat cells with stable TCERG1 mRNA interference. Jurkat cell lines were constructed by transfection of pGeneClip-shTCERG1 vectors. Jurkat-shTCERG1-(3-4) and Jurkat-shTCERG1-(1-3-4) cell lines were generated by co-transfection with pGeneClip-shTCERG1-3 and pGeneClip-shTCERG1-4 and/or pGeneClip-shTCERG1-1 vectors. The control Jurkat-shTCERG1-C1 cell line was generated by transfection with the pGeneClip-shTCERG1-1 vector. All of the cell lines were tested for shRNA-mediated TCERG1 depletion by semiquantitative RT-PCR assay. Jurkat-shTCERG1-(3-4) and Jurkat-shTCERG1-(1-3-4) cell lines showed approximately 70% and 80% TCERG1 depletion, respectively (Figure 1B). In agreement with our previous data, we observed significantly reduced basal and Tat-activated transcription from the HIV-1 LTR upon TCERG1 knockdown in these Jurkat cells (Figure 1B). We noted that the inhibition of transcription was reduced in the presence of Tat in both cell systems (compare right and left panels in Figure 1). This result might indicate that TCERG1 acts on basal transcription rather that on Tat-mediated *trans*-activation. Immunofluorescence analysis indicated that TCERG1 has no effect on the spatial localization of Tat (Figure 1B). Together, these findings indicate that TCERG1 depletion affects basal and Tat-mediated transcriptional activation of the HIV-1 LTR in lymphoid cells. We conclude that TCERG1 plays a positive role in efficient transcription from the HIV-1 LTR.

### TCERG1 depletion impairs elongation of HIV-1 transcripts

**Figure 2:**
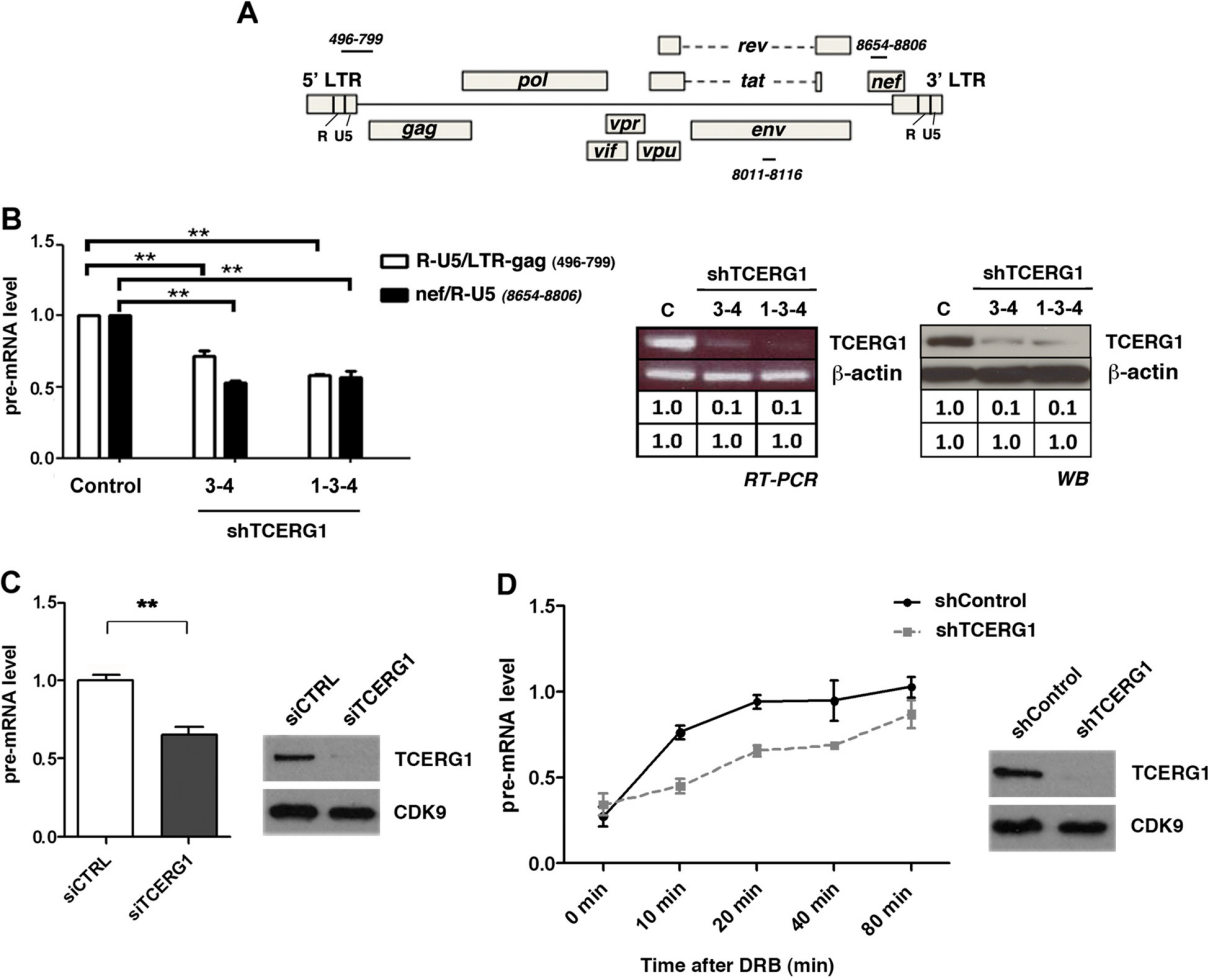
**TCERG1 depletion impairs elongation of HIV-1 transcripts. A.** Schematic representation of the HIV-1 genome. The black lines indicate the position of each pair of primers used for qPCR experiments. **B.** Analysis by qPCR of the amount of nascent transcripts originated at the proximal (LTR-gag) and distal (nef) regions of the pNL4-3-wt strain in Jurkat cells with stable interference of TCERG1 (left panel). The results of three different experiments are represented in the histogram (means ± SEM; **, *p <* 0.01). TCERG1 mRNA interference was assessed by semi-qPCR and immunoblotting using β-actin as the housekeeping gene and loading control, respectively (right panels). **C.** Quantitative analysis of the amount of nascent transcripts originated at distal regions of the pNL4-3∆RT provirus. HEK293T cells were transfected with siRNAs against EGFP (siCTRL) or TCERG1 (siTCERG1) together with the pNL4-3∆RT plasmid and pXGH5 as a transfection control. Total RNA was extracted and qRT-PCR was performed using primers that amplify the *env* gene of the provirus (at positions 8010-8116). The graph shows the data from triplicates of three independent experiments as values relative to those obtained from cells without siRNAs transfectiona (means ± SEM; **, *p* < 0.01). **D.** Kinetics of RNAPII-dependent transcription elongation in the presence or absence of TCERG1. Control and TCERG1-knockdown T-Rex-HEK293 cells were transfected with pNL4-3∆RT and pXGH5 as a transfection control and treated 48 h later with 100 μM DRB for 3 h. qRT-PCR was performed using the primers that amplify the *env* gene of the provirus. The graph shows pre-mRNA levels at different times from the control and TCERG1-depleted cells. The data shown are the averages from triplicates of three independent experiments (mean ± SEM). The analysis of TCERG1 knockdown in T-Rex-HEK293 cells by Western blotting is shown on the right. CDK9 is used as loading control.

To test whether TCERG1 affects HIV-1 transcriptional elongation, we measured the amount of transcripts generated at proximal and distal regions of the HIV-1 gene. We transfected the Jurkat cell lines with the pNL4-3-wt viral clone and assessed the amount of viral transcripts by qRT-PCR. We designed specific primers directed against the R/U5-gag and env/nef regions (Figure 2A). We found that both early and late elongation of viral transcripts was inhibited by approximately 50% in the Jurkat-shTCERG1 cell lines (Figure 2B, left panel). To lend support to the observation that TCERG1 affects elongation, we measured the pre-mRNAs generated at distal regions from a transiently transfected plasmid that contains the complete pro-virus genome with a deletion in the retrotranscriptase protein (pNL4-3ΔRT) under conditions of TCERG1 knockdown in HEK293T cells. We performed these experiments in HEK293T cells because we have previously observed that TCERG1 affects the level of elongation-competent transcription complexes in these cells [50]. The pNL4-3ΔRT DNA generates viral particles in HEK293T cells that are able to enter target cells, but due to the absence of reverse transcriptase, no replication is detected. The cDNA was synthesized using random hexamers followed by qPCR with primers amplifying a region corresponding to the *env* gene (Figure 2A). A plasmid expressing human growth hormone (pXGH5) was also transfected and amplified by qPCR as a transfection control (data not shown). The levels of RNA were normalized to those of endogenous GAPDH. Similarly with the data obtained in Jurkat cells (Figure 2B), we observed a diminished accumulation of distal transcripts by approximately 30-40% upon TCERG1 knockdown (Figure 2C, left panel). The level of TCERG1 expression in silenced cells compared to that of CDK9 is also shown (Figure 2C, right panel). This result confirms the previous data and further suggests that TCERG1 acts through an elongation mechanism in the regulation of HIV-1 gene expression.

To directly test whether TCERG1 affects the rate of RNAPII transcription, we used Padgett’s protocol. In this procedure, 5,6-Dichlorobenzimidazole 1-β–D-ribofuranoside (DRB), which reversibly blocks gene transcription *in vivo* by inhibiting the P-TEFb-dependent Ser 2 phosphorylation of the CTD of RNAPII, is used in combination with qRT-PCR to analyze the transcription of human genes [54]. To perform this protocol under optimal conditions, we used T-Rex-HEK293 cell lines, which ensure high transfection efficiency, in which expression of control shRNA and shRNA targeting TCERG1 can be induced by addition of tetracycline. The analysis of cell lysates from induced cells showed that the cells that contain shRNAs targeting TCERG1 express significantly lower TCERG1 protein levels compared to the control shRNAs (Figure 2D, right panel). Control and TCERG1-knockdown cells were transfected with pNL4-3ΔRT and pXGH5. The cells were treated with DRB, and samples were collected at different time points after DRB removal. Quantitative RT-PCR was performed using the primers indicated above to amplify a region corresponding to the *env* gene (Figure 2A). The DRB-treated control cells were able to recover transcription within 20 to 80 min after DRB removal, which is consistent with a transcriptional lag due to the genomic distance from the start site of transcription [54]. In contrast, transcriptional recovery in TCERG1-knockdown cells was significantly slower (Figure 2D, left panel). These results demonstrate a role for TCERG1 in the RNAPII transcription of HIV-1 *in vivo*.

### TCERG1 is present at the promoter and coding regions of the HIV-1 gene

**Figure 3:**
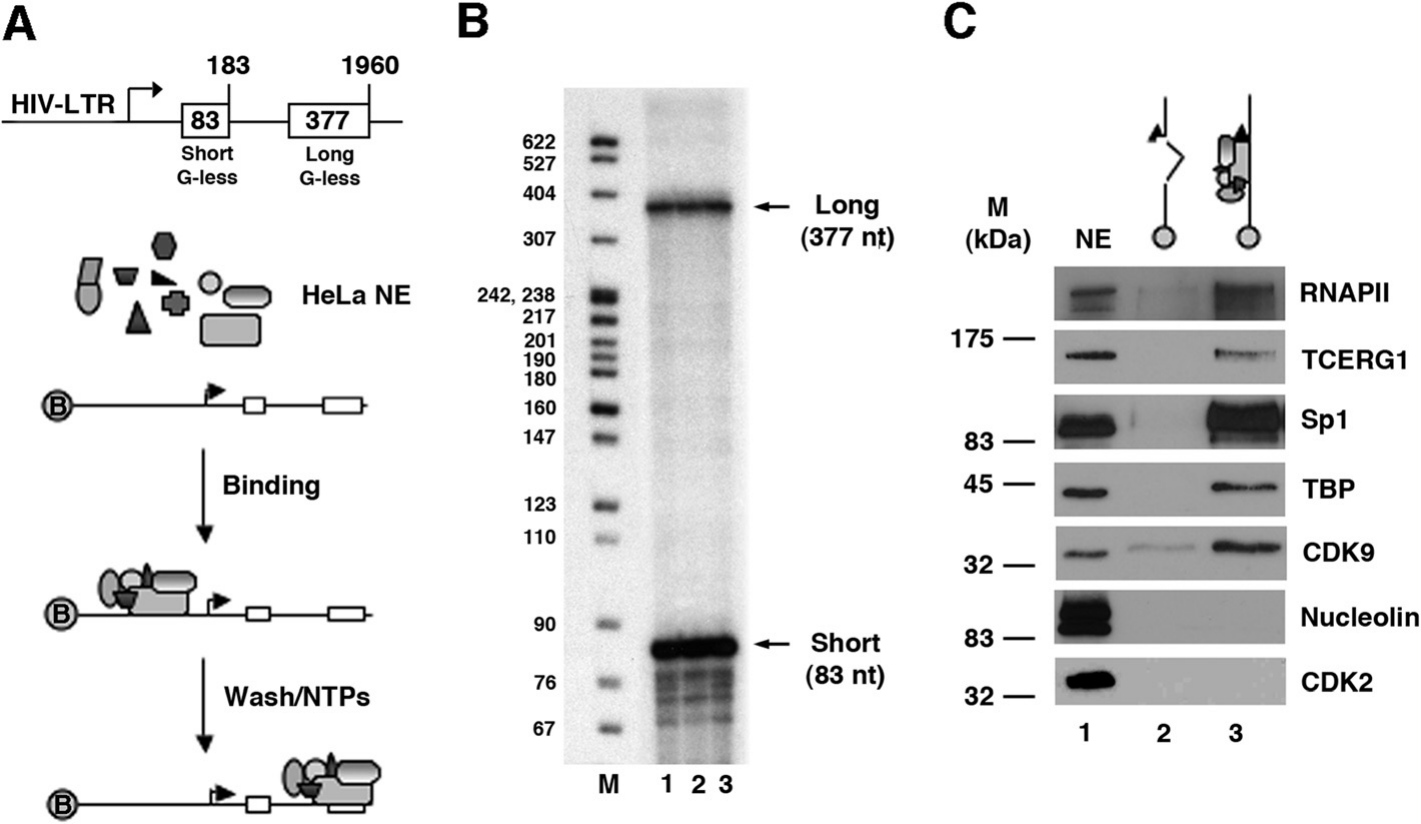
**Analysis of PICs assembled on the HIV-1 promoter *in vitro. *A.** Experimental strategy for analyzing active PICs. Step 1: Cell-free transcription reactions are performed using HeLa nuclear Extract (NE) and biotinylated templates carrying the HIV-1 LTR. Step 2: Transcription complexes are purified by binding to streptavidin-coated magnetic beads. Step 3: Nascent RNA chains are labeled by incorporation of α-[^32^P]UTP. A schematic representation of the HIV-1 double G-less cassette template used in the experiment is shown at the top of the panel. **B.***In vitro* transcription reactions were performed with the purified PICs. Lanes 1-3 represent three independent reactions performed using the HIV-1 template. Arrows indicate the migration of long and short transcripts. **C.** TCERG1 is a component of the HIV-1 PICs. PICs were formed on constructs containing (lane 3) or not (lane 2) the HIV-1 promoter. PICs were purified with streptavidin-coated magnetic beads, and Western blot analysis of the purified PICs was performed using the specific antibodies shown at the right side of the panel. The relative mobilities (in kDa) of the molecular mass markers (M) are shown on the left side of the panel.

The above functional results and other published data [51,53,55,56] indicate that TCERG1 associates with components of the transcriptional machinery to regulate HIV-1 transcriptional elongation. The point at which TCERG1 associates with the transcriptional complex during HIV-1 transcriptional activation remains an important question in understanding TCERG1 function. To address this issue, we used a combination of *in vitro* and *in vivo* approaches. First, to directly measure the transcribing RNAPII in an *in vitro* transcription assay, we used an immobilized template consisting of a biotinylated double G-less cassette template driven by the HIV-1 LTR to isolate HIV-1 RNAPII pre-initiation complexes (PICs) [35]. This template synthesizes transcripts that contain two regions (cassettes) of different sizes that lack guanosine residues; therefore, these G-less cassettes are resistant to digestion with RNase T1. One G-less cassette is located proximal to the promoter, enabling measurement of the numbers of transcription complexes that reach nucleotide +183 (short), while the second downstream G-less cassette measures the number of transcripts beyond nucleotide +1960 (long) (see Figure 3A). The biotinylated templates were incubated with HeLa nuclear extract and subsequently isolated using streptavidin-coated magnetic beads. We performed *in vitro* transcription assays to test the activity of the isolated transcription complexes. We observed clear signals for the short and long transcripts (Figure 3B), which indicated that the isolated complexes were competent to transcribe the DNA sequences. We next analyzed the relative protein composition of the PICs formed on the HIV-1 promoter sequences, especially with respect to TCERG1. Figure 3C shows a representative immunoblot analysis of PICs using RNAPII-, TCERG1-, Sp1-, TBP-, CDK9-, nucleolin-, and CDK2-specific antibodies. As expected, RNAPII, Sp1, TBP, and CDK9 were detected in the PICs formed on the HIV-1 promoter. Interestingly, TCERG1 was also found in the PICs. We did not detect binding of nucleolin or CDK2 to the PICs. These results demonstrate the presence of TCERG1 in the RNAPII complexes assembled onto the HIV-1 promoter *in vitro*.

**Figure 4:**
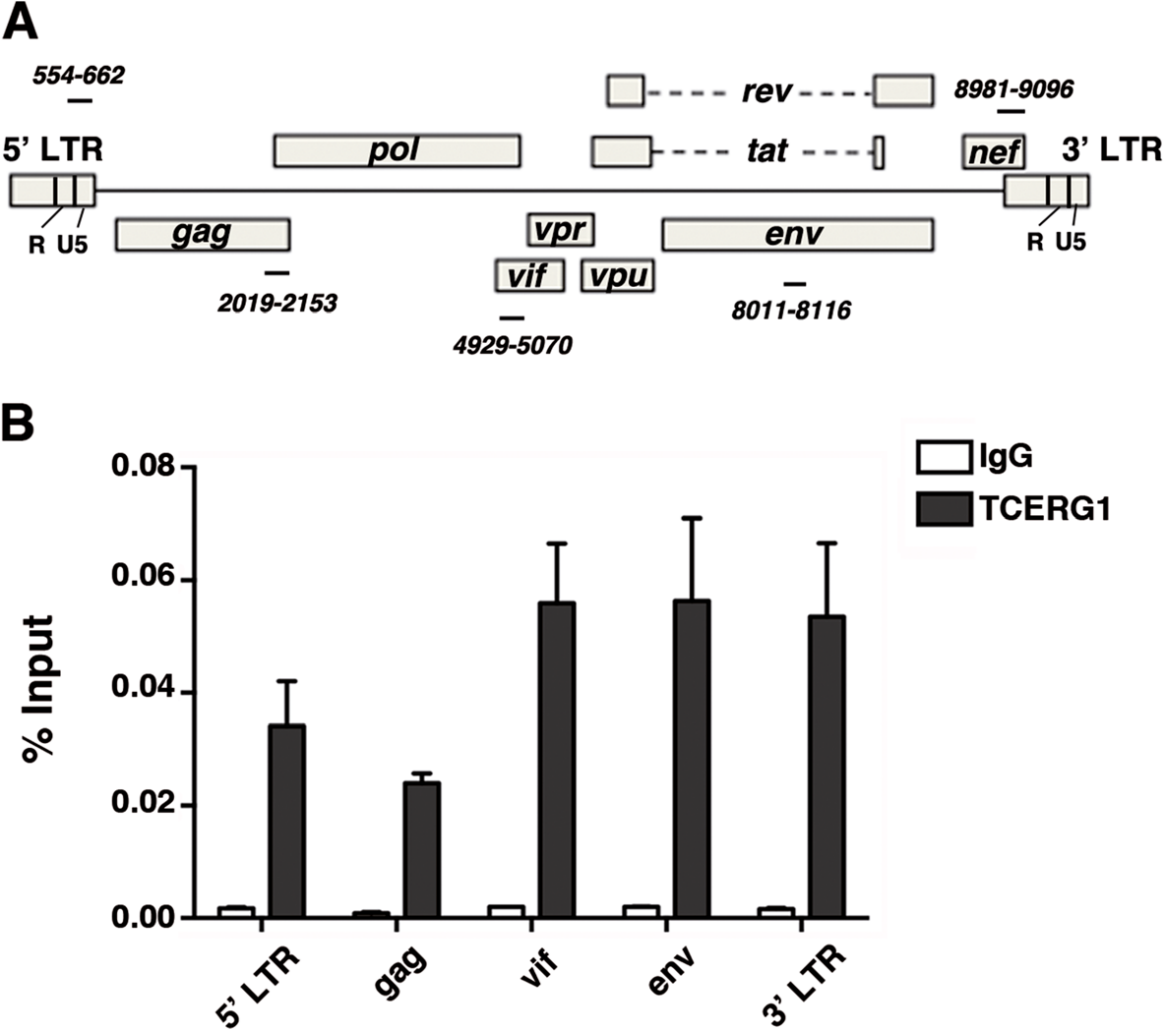
**TCERG1 binds along the HIV-1 genome. A.** Schematic representation of the HIV-1 genome. The black lines indicate the position of each pair of primers used for qChIP experiments. **B.** TCERG1 distribution at different positions on the HIV-1 gene detected by ChIP followed by qPCR in HEK293T cells transfected with the pNL4-3∆RT provirus and a TCERG1 overexpression vector. IgG was used as a control in all ChIP experiments. Data from four independent experiments are presented as percentages of the bound input.

Next, we determined whether transiently transfected TCERG1 is recruited to the HIV-1 LTR *in vivo* by measuring the density of TCERG1 at specific regions of the pNL4-3ΔRT gene using quantitative ChIP (qChIP) analysis. We designed specific primers to detect TCERG1 recruitment at the 5′ LTR, gag, vif, env, and 3′ LTR regions of the HIV-1 gene (Figure 4A). The qChIP experiments revealed a specific association of TCERG1 at all these regions (Figure 4B). TCERG1 was not detected using control IgG antibodies or control intergenic regions (Figure 4 and data not shown). These results indicate that TCERG1 specifically associates with both, the promoter and downstream regions of the HIV-1 gene *in vivo*.

### TCERG1 depletion decreases phosphorylation of Ser2 within the RNAPII CTD

**Figure 5:**
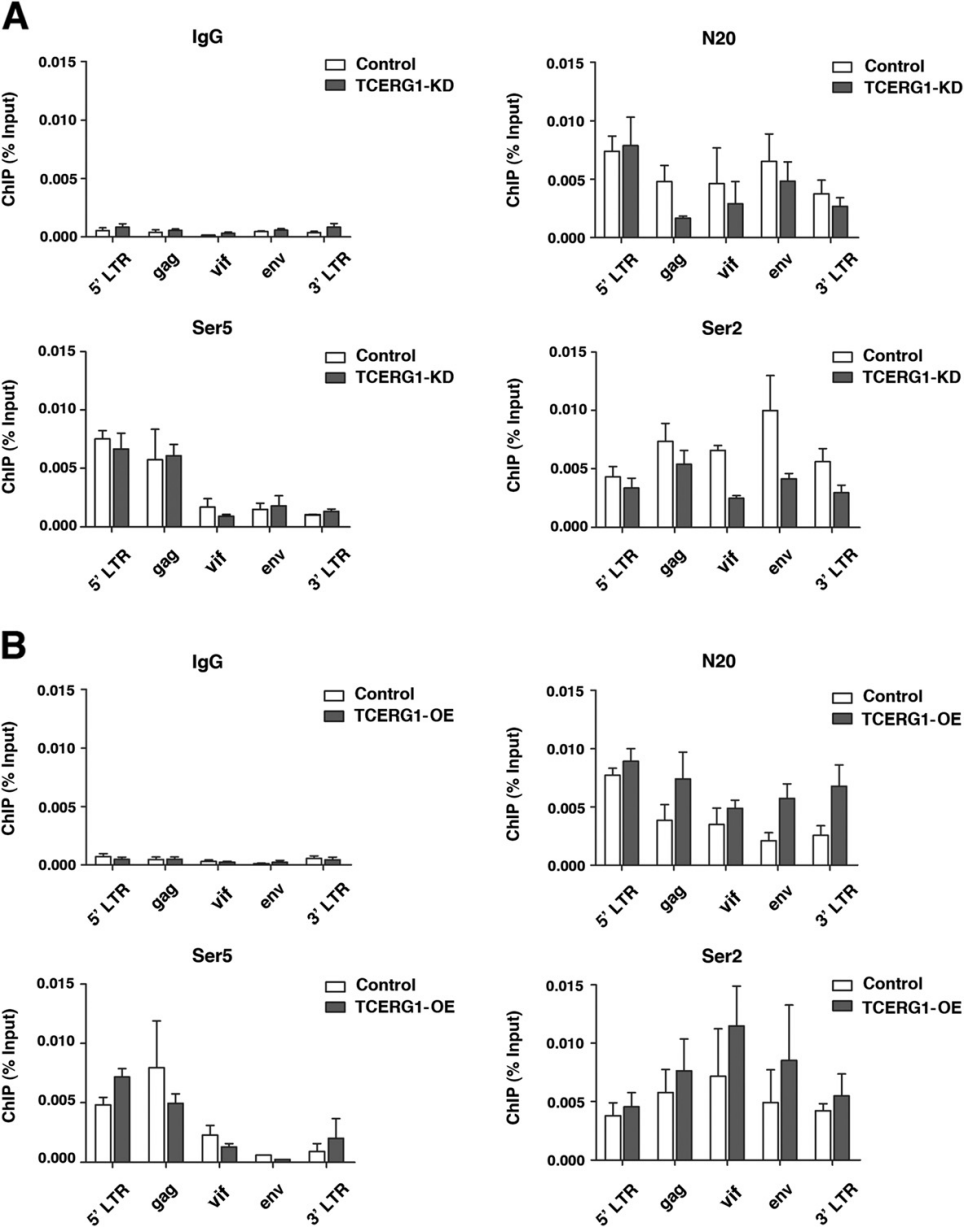
**TCERG1 depletion decreases CTD Ser2 phosphorylation. A.** Total polymerase (N20), phospho-Ser5 (Ser5) or phospho-Ser2 (Ser2) polymerase II distribution at different positions of the HIV-1 genome was detected by ChIP followed by qPCR in the control and TCERG1 knockdown (KD) T-Rex-293 cell lines transfected with the pNL4-3ΔRT provirus. **B.** The same experiment described in panel **A** was performed using empty (control) and TCERG1 overexpression (OE) vectors. IgG was used as a control in all ChIP experiments. Data from four independent experiments are presented as percentages of the bound input (means ± SEM).

The above results demonstrate a specific association of TCERG1 along the HIV-1 genome that resembles the pattern of the RNAPII recruitment onto a gene unit. In addition, transcriptional elongation is known to be dependent on RNAPII CTD phosphorylation along the transcribed gene [57,58]. Because TCERG1 has previously been shown to bind to the phosphorylated CTD of RNAPII [52], we examined whether the observed decrease in the elongation rate of the enzyme (Figure 2D) was due to changes in the CTD phosphorylation pattern. To perform these experiments, we analyzed the phosphorylation of the RNAPII CTD under conditions of TCERG1 knockdown or overexpression. We measured the density of RNAPII at the 5′ LTR, gag, vif, env, and 3′ LTR regions of a transiently transfected pNL4-3ΔRT provirus (see Figure 4A) by qChIP analysis using specific antibodies against total RNAPII (N20), Ser2, and Ser5 (Figure 5). TCERG1 depletion resulted in a decreased accumulation of total RNAPII and RNAPII phosphorylated at Ser2, which was more pronounced at the distal positions of the gene (Figure 5A). No differences were observed at the promoter region upon TCERG1 knockdown. The recruitment of RNAPII phosphorylated at Ser5 also remained unaltered (Figure 5A). Conversely, TCERG1 overexpression resulted in an accumulation of total and Ser2 polymerases at the same positions (Figure 5B). These results indicate that TCERG1 is able to modify CTD phosphorylation at Ser2, which may explain how TCERG1 affects the dynamics of HIV-1 transcript elongation.

TCERG1 did not change the overall amount of Ser2 phosphorylation or total RNAPII in the cell upon TCERG1 depletion as assessed by immunoblotting analysis (Additional file 1: Figure S1A). Compensatory mechanisms to prevent loss of a protein as important as the RNAPII may occur in the stable transfectant cells. We detect a slight increase in the amount of Ser2 phosphorylation and total RNAPII upon TCERG1 overexpression (Additional file 1: Figure S1B). The latter result is in agreement with our ChIP data but caution must be taken in interpreting these results because we are measuring global Ser2 phosphorylation in the cell and our ChIP experiments of Figure 5 are restricted to the recruitment of RNAPII on the HIV-1 genome.

### TCERG1 is required for HIV-1 replication

**Figure 6:**
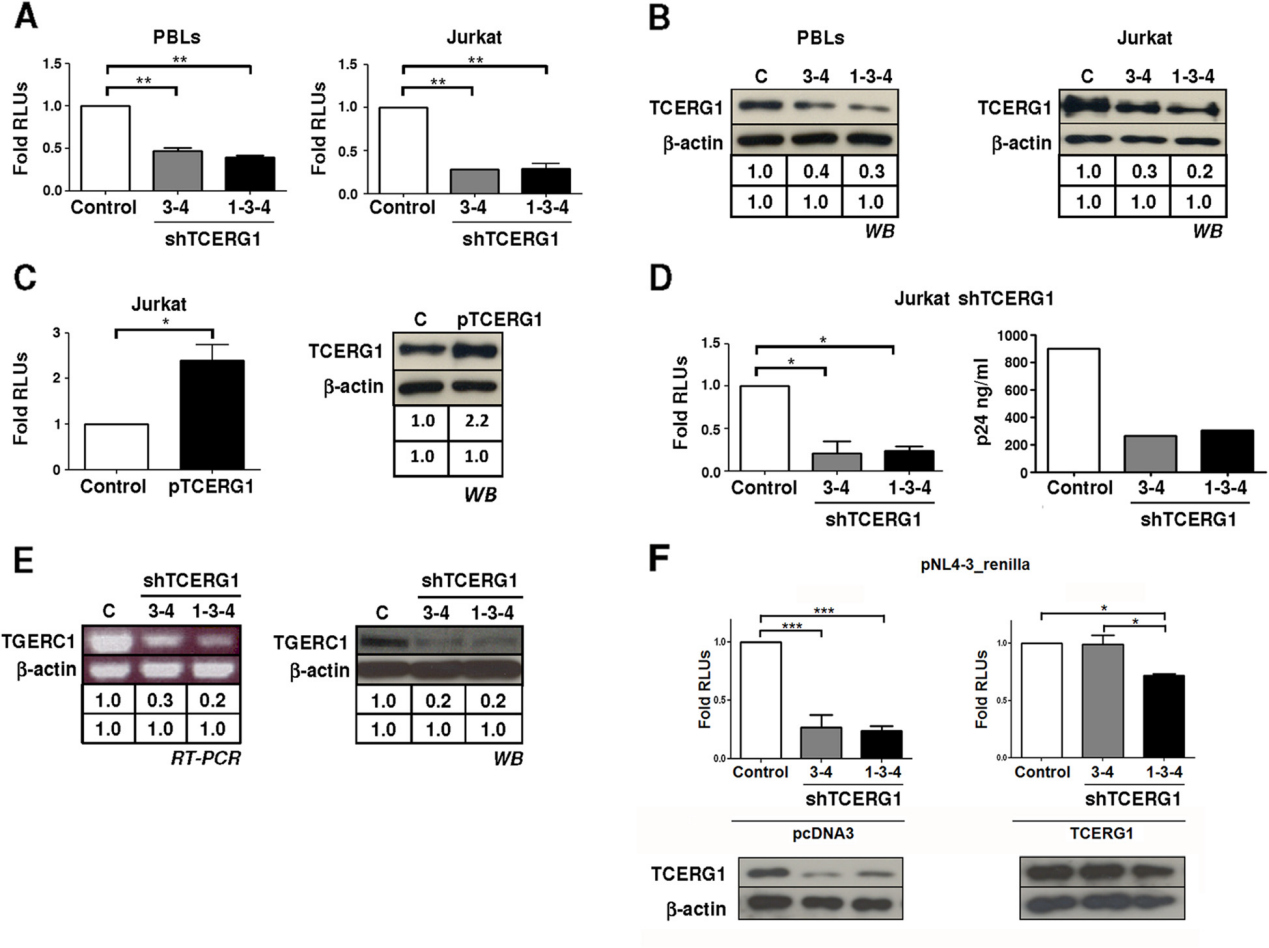
**TCERG1 is required for HIV-1 replication. A.** PBLs and Jurkat cells were transiently transfected with the infectious clone pNL4-3-Renilla along with pGeneClip-shTCERG1-C1 or pGeneClip-shTCERG1-3 and pGeneClip-shTCERG1-4, and the latter pair together with pGeneClip-shTCERG1-1. Renilla was measured 72 hours after transfection, and RLUs are expressed relative to the control cells. **B.** Expression of TCERG1 protein was assessed by Western blot in both PBLs and Jurkat. **C.** Jurkat cells were co-transfected with pNL4-3-Renilla and the pEFBOST7-TCERG1 expression vector or empty pcDNA3 as the basal control. Overexpression of TCERG1 was assessed by Western blot. **D.** Jurkat cells with stable TCERG1 mRNA interference (Jurkat shTCERG1-(3-4) and Jurkat shTCERG1-(1-3-4)) were transfected with pNL4-3-Renilla. Renilla activity was measured in the infected cells (left panel), and p24 was measured in the culture supernatants (right panel); the data are expressed relative to the control cells. **E.** The TCERG1 interference was analyzed in Jurkat shTCERG1-(3-4) and Jurkat shTCERG1-(1-3-4) by semiquantitative RT-PCR and Western blot. **F.** The rescue of HIV-1 replication in Jurkat cells with stable TCERG1 mRNA interference (Jurkat shTCERG1-(3-4) and Jurkat shTCERG1-(1-3-4)) transfected with pNL4-3_renilla vector was assessed by co-transfection with a TCERG1 expression vector (right panel), in comparison with cells transfected with pcDNA3 as negative control (left panel). Renilla activity was measured and represented as RLUs; the data are expressed relative to the control cells. The TCERG1 interference and overexpression was determined by Western blot. In all cases, the data from three different experiments is represented in the histograms (mean ± SEM). Statistical analyses were performed and are shown as *, *p <* 0.05; **, *p <* 0.01; ***, *p <* 0.001.

Although previous experiments have implicated TCERG1 in HIV-1 LTR regulation using transient transfection approaches, previous reports have not addressed the functional implications of these findings on viral replication *in vivo*. Therefore, we conducted *in vivo* assays to test whether TCERG1 can regulate HIV-1 replication. PBLs isolated from healthy donors or Jurkat cells were co-transfected with the HIV-1 infectious clone pNL4-3-Renilla along with the pGeneClip-shTCERG1-C1 or pGeneClip-shTCERG1-3 and pGeneClip-shTCERG1-4 and/or pGeneClip-shTCERG1-1 vectors. After 18 h, the cells were collected and lysed, and Renilla luciferase activity was measured. TCERG1 knockdown caused a more than 2-fold reduction in HIV-1 replication in PBLs and a more than 3-fold reduction in Jurkat cells (Figure 6A). The TCERG1 interference was analyzed by Western blot in both PBLs and Jurkat; β-actin was used as the loading control (Figure 6B). The co-transfection of a TCERG1 expression vector and the HIV-1 infectious clone pNL4-3-Renilla produced the opposite effect on HIV-1 replication with a more than a 2-fold increase in the synthesis of Renilla in Jurkat cells (Figure 6C, left panel). Overexpression of TCERG1 was assessed by Western blot (Figure 6C, right panel). To lend additional support to our data, Jurkat-shTCERG1 cell lines were transfected with the HIV-1 infectious clone pNL4-3-Renilla. HIV-1 replication was reduced in both Jurkat-shTCERG1-(3-4) and Jurkat-shTCERG1-(1-3-4) cell lines (Figure 6D, left panel). Finally, to corroborate the notion that the observed inhibition is a consequence of diminished viral replication, we measured the expression of the p24 viral capsid protein. Measurement of HIV-1 p24 antigen levels is a highly specific assay for monitoring HIV-1 replication and the progression of HIV-1 infection [59] and correlates with the expression of Renilla [60]. We found that expression of p24 was inhibited by approximately 75% in TCERG1-silenced Jurkat cells compared to control cells (Figure 6D, right panel). TCERG1 mRNA and protein interference were analyzed by RT-PCR and Western blot, respectively (Figure 6E). These data further support the results of our previous experiments indicating that TCERG1 is indeed required for HIV-1 replication *in vivo*. To lend additional support and specificity to our data, we tested the effect of overexpressing TCERG1 on HIV-1 replication in the Jurkat-shTCERG1 cell lines. Transient overexpression of TCERG1 in TCERG1 knockdown cells rescued HIV-1 replication (Figure 6F). Given that Tat recruits the P-TEFb complex to the RNA TAR element and activates transcription elongation through phosphorylating the RNAPII CTD, we also sought to assess the effect of CDK9 on TCERG1 depletion. To this goal, we used expression plasmids for CDK9 and a catalytically inactive CDK9 protein bearing a single amino acid change (Asp-167 to Asn) in its active domain (CDK9dn). Previous data have shown that CDK9dn is functionally able to associate *in vitro* and *in vivo* with Cyclin T [61]. And in Jurkat cells, overexpression of Cdk9dn specifically inhibited Tat *trans*-activation and HIV-1 replication [62] by preventing the endogenous CDK9 protein from interacting with CyclinT1. Importantly, CDK9dn fails to activate viral transcription indicating that the kinase activity of CDK9 is essential for transcription activation [35,63]. Our experiments with CDK9/CDK9dn were designed to prove that our observations were specific for HIV-1 and it could compensate (or not) for a lack of TCERG1. Therefore, the experiments were performed with a reporter LTR-HIV plasmid in order to analyze the transcriptional effects of the proteins avoiding the dominant negative effect of CDK9dn. Overexpression of CDK9 did not rescue the LTR-dependent transcription in the absence of endogenous TCERG1 (Additional file 2: Figure S2).

## Discussion

Our results help to explain the mechanism by which TCERG1 functions in HIV-1 replication. We report that TCERG1 is required for both basal and Tat-dependent transcriptional activation in cells of the immune system, which are the relevant targets in HIV-1 infection. Previous reports have indicated that immunodepletion of TCERG1 from transcriptionally-active HeLa nuclear extracts decreases Tat activation of RNAPII elongation efficiency *in vitro*. However, TCERG1 does not bind directly to Tat, and the presence of TCERG1 in the Tat affinity column was later suggested to be due to the association of TCERG1 with other HIV-1 cofactors, such as P-TEFb and Tat-SF1 [51]. Based on this possibility, it was conceivable that removal of the TCERG1-associated Tat cofactors, not removal of TCERG1 per se, provoked the decrease in Tat-mediated activation of transcriptional elongation. Here, we show that depletion of TCERG1 by gene silencing resulted in decreased basal and Tat-activated transcription, thus demonstrating a direct effect for TCERG1 in the regulation of HIV-1 transcription. We show previously that TCERG1 affects elongation in a promoter-specific fashion [50,53] and a relatively small number of genes are affected upon TCERG1 depletion by microarray analysis [64]. Moreover, loss of *tcer-1*, the TCERG1 homologue in *C. elegans*, has little or no effect on wild-type lifespan [65]. Thus TCERG1 is not likely to be a general component of the transcriptional machinery, therefore, the most probable interpretation of our experimental data is that TCERG1 is affecting specifically to HIV-1 transcription. However, we cannot rule out the possibility of TCERG1 affecting cellular genes that are important for viral transcription.

The comparison of our results with those observed for the other HIV-1 Tat cofactor, Tat-SF1 (which was also identified by *in vitro* reconstituted transcription reactions with immunodepleted extracts), is interesting. In recent work, we show that depletion of Tat-SF1 by gene silencing did not affect basal or Tat-dependent transcription from an HIV-1 CAT reporter in HeLa and HEK293 cells [66]. These data contradict the results obtained by Caputi and coworkers, which have recently reported that down-regulation of Tat-SF1 by siRNAs induces a decrease in transcription and Tat-mediated activation of an HIV-1 reporter minigene in HEK293 cells [67]. In this later work, the effect of reduction of TCERG1 expression was also analyzed. In contrast with our results, inhibition of TCERG1 expression partially reduced Tat-mediated *trans*-activation by paradoxically increasing the basal transcription of the reporter construct [67]. Our experiments with TCERG1 were predominantly performed in Jurkat and PBLs, which are often used to examine transcriptional regulation and HIV-1 infection in lymphocytes, the natural targets of HIV-1. In agreement with the results obtained in these cells, we also observed inhibition of transcription from a proviral vector upon depletion of TCERG1 in HEK293 cells (Figure 2C, Figure 2D, and other data not shown). Therefore, caution must be taken in interpreting the results obtained with Tat-SF1 and TCERG1 knockdown since different results can be obtained in different contexts. Other elements in the HIV-1 genome, the cell line used, and the chromatin environment created by the different HIV-1 plasmids could potentially contribute to the proper recruitment and functionality of these Tat cofactors.

What is the mechanism for the decrease in HIV-1 transcription upon TCERG1 depletion? Our results provide a mechanism that is based on the RNAPII elongation rate. First, we observed diminished accumulation of transcripts upon TCERG1 knockdown (Figure 2B and Figure 2C). Second, TCERG1 increased the rate of RNAPII transcription (Figure 2D). Finally, we observed that TCERG1 favors the phosphorylation of Ser2 of the RNAPII CTD (Figure 5). Based on these results, we propose that TCERG1 regulates HIV-1 transcription by increasing the phosphorylation of Ser 2 on the heptad repeats in the CTD, which facilitates the elongation of RNAPII through the DNA template during gene activation. We have taken several approaches to assess the recruitment of TCERG1 to the HIV-1 gene. Isolation of PICs assembled onto a functional HIV-1 promoter *in vitro* identified the presence of TCERG1 (Figure 3). ChIP analysis confirmed the presence of TCERG1 at the promoter region and distal elements (Figure 4). The recruitment of TCERG1 correlates with the modulation of Ser2 phosphorylation upon TCERG1 knockdown/overexpression (Figure 5). These results are consistent with a role for TCERG1 in the activation of HIV-1 transcriptional elongation that leads to the release of RNAPII from promoter-proximal pausing. However we cannot exclude the possibility that TCERG1 favors RNAPII processivity by acting at other locations within the gene unit where polymerases could pause.

Very recently, a multi-component complex now called the super elongation complex (SEC) has been isolated through a sequential affinity-purification strategy to identify proteins associated with both Tat and P-TEFb [68]. In addition to Tat and P-TEFb, the SEC contains the transcription factors ELL2, AFF4, ENL, and AF9. The combined action of Tat, P-TEFb and the SEC results in the synergistic activation of HIV-1 elongation by helping to release RNAPII from promoter-proximal pausing and promote the production of full-length viral transcripts [68,69]. ELL2 is a well-characterized transcription elongation factor and member of the ELL family of transcription factors that stimulate elongation by increasing the catalytic rate and suppressing transient pausing of RNAPII [70,71]. Strikingly, we have identified ELL as a TCERG1-interacting protein using a tandem affinity purification (TAP) strategy (data not shown). Our data show that overexpression of CDK9 did not rescue the HIV-1 LTR-dependent transcription in the absence of endogenous TCERG1 (Additional file 2: Figure S2). Based on these observations, it is an exciting possibility that TCERG1 affects RNAPII processivity via a mechanism that involves the recruitment and/or assembly of functional transcription complexes.

One novel finding presented here is that TCERG1 is required for HIV-1 replication (Figure 6). This requirement is likely due to the effect of TCERG1 on viral transcription, although the precise mechanism by which this factor functions remains unknown. Early data that suggested a role for TCERG1 in HIV-1 replication found that the levels of TCERG1 increased in Jurkat T cells cultured with CXCL12 or the HIV-1 glycoprotein gp120 IIIB [72]. This expression was found to be dependent on the presence of the HIV-1 co-receptor CXCR4 [72]. Recently, several studies using genome-wide RNAi experiments have identified the so-called HIV dependency factors (HDFs) [73-75]. TCERG1 was not identified in any of these studies. However, the three siRNA screens showed little overlap at the level of individual genes, perhaps due to differences in the cell lines and HIV strains used, the assay time post-infection or the procedures used to measure infection. A recent work exploiting the interactions identified in these RNAi screens in the context of a human protein-protein interaction network has predicted undiscovered HDFs that may play a role in HIV-related disease progression in lymph nodes. Importantly, TCERG1 was one of the newly predicted HDFs in this study [76].

## Conclusions

Although we have gained a considerable knowledge regarding the early events that occur at the promoter region of genes to trigger the release of RNAPII for elongation, several recent discoveries have suggested that events subsequent to transcript initiation may also be necessary for the production of full-length transcripts. Specifically, the rate of elongation and pausing of RNAPII are generating considerable interest as critical regulators to ensure precise and reliable control of gene expression. In this scenario, factors that affect these processes could act as checkpoint regulators to guarantee optimal RNAPII activity. In this work, we describe the nuclear factor TCERG1 as positive regulator of HIV-1 transcriptional elongation by increasing the elongation rate of RNAPII and phosphorylation of Ser 2 within the CTD. TCERG1 is therefore a candidate checkpoint factor that could mediate functional links between elongation rates and pausing.

The experiments reported here demonstrate an important role for TCERG1 in controlling HIV-1 transcriptional elongation and replication. These studies suggest that TCERG1 might be a plausible new target for anti-retroviral therapy, thus opening interesting new avenues for future investigation.

## Methods

### Plasmids

The pNL4-3 wild-type (wt) plasmid, which contains the complete HIV-1 genome and induces infectious progeny after transfection, was kindly provided by Dr M.A. Martin [77]. The pNL4-3-ΔRT plasmid was created by deleting the reverse transcriptase gene of the pNL4-3 wt plasmid and was kindly provided by Dr Sánchez-Palomino (Hospital Clínic, Barcelona) [78]. The pNL4-3-Renilla plasmid was obtained by replacing the *nef* gene in the HIV-1 proviral clone pNL4-3 with the Renilla luciferase gene, as previously described [60]. The LTR-LUC plasmid containing the luciferase (LUC) reporter gene under the control of the HIV-1 LTR U3 + R region (LAI strain) has previously been described [79]. The pCMV-Tat101 plasmid has previously been described [80]. The eukaryotic expression pEFBOST7-TCERG1 plasmid has been previously described [50]. CDK9 and CDK9dn expression plasmids were described previously [35]. The GeneClip U1 Hairpin Cloning System kit (Promega Biotech Iberica, Madrid, Spain), which contains the pGeneClip vector, was used to generate the following small hairpin RNA (shRNA) plasmids: pGeneClip-shTCERG1-1, pGeneClip-shTCERG1-3, and pGeneClip-shTCERG1-4) containing three different small interference RNA (siRNA) sequences directed against the mRNA encoding for TCERG1. The sequences used to generate the pGeneClip-shTCERG1-1 plasmid were shTCERG1-1 s (5′-TCTCGAAGGAGTTGCACAAGATAAAGTTCTCTTATCTTGTGCAACTCCTTCCT-3′) and shTCERG1-1 as (5′-CTGCAGGAAGGAGTTGCACAAGATAAGAGAACTTTATCTTGTGCAACTCCTTC-3′). The sequences used to generate the pGeneClip-shTCERG1-3 plasmid were shTCERG1-3 s (5′-TCTCGATCCTCGATGTATTAAGTAAGTTCTCTACTTAATACATCGAGGATCCT-3′) and shTCERG1-3as (5′-CTGCAGGATCCTCGATGTATTAAGTAGAGAACTTACTTAATACATCGAGGATC-3′). The sequences used to generate the pGeneClip- shTCERG1-4 plasmid were shTCERG1-4 s (5′- TCTCGGCATGACTGACATACATAAAGTTCTCTTATGTATGTCAGTCATGCCCT-3′) and shTCERG1-4as (5′-CTGCAGGGCATGACTGACATACATAAGAGAACTTTATGTATGTCAGTCATGCC-3′). The scrambled sequences used to generate the pGeneClip-shTCERG1-C1 plasmid were shTCERG1-C1s (3′-TCTCGGACTGGAAGTTCAAAGAAAAGTTCTCTTTCTTTGAACTTCCAGTCCCT-5′) and shTCERG1-C1as (3′-CTGCAGGAGAGGCGTTTTAGAATATAGAGAACTTATATTCTAAAACGCCTCTC-5′). Each pair of primers were annealed and cloned in the linearized pGeneClip vector according to the manufacturers’ instructions. The pEGFP-C1 vector, which was used as control for monitoring transfection efficiency, was purchased from Clontech (Mountain View, CA). Plasmids were purified using the QIAGEN Plasmid Maxi Kit (Qiagen Iberia, Madrid, Spain) following the manufacturer’s instructions. The human growth hormone-expressing plasmid pXGH5 was kindly provided by Dr Heiner Schaal (University of Düsseldorf, Germany). The pEYFP-C1 vector (BD Biosciences, Clontech) was co-transfected as a control for monitoring transfection efficiency and was measured using a FACSCalibur flow cytometer (BD Biosciences, Clontech).

### Cells

The Jurkat cell line was cultured in RPMI 1640 medium (BioWhittaker, Walkersville, MD) supplemented with 10% fetal calf serum (PAN Biotech GmbH, Aidenbach, Germany), 2 mM L-glutamine, 100 μg/ml streptomycin and 100 U/ml penicillin (Lonza, Basel, Switzerland) at 37°C. PBMCs were isolated from the blood of at least three different healthy donors by centrifugation through a Ficoll-Hypaque gradient (Lymphocyte Separation Medium, Lonza). The adherent monocytes were eliminated by keeping the culture flask horizontal for 1 hour and then separating the non-adherent lymphocytes. These peripheral blood lymphocytes (PBLs) were collected in supplemented RPMI 1640 medium and maintained at 2 × 10^6^ cells/ml at 37°C.

Two Jurkat cell lines with stably interference for TCERG1 were produced: Jurkat shTCERG1-(3-4), which was co-transfected with both plasmids pGeneClip-shTCERG1-3 and pGeneClip-shTCERG1-4 (1:1); and Jurkat shTCERG1-(1-3-4), which was co-transfected with plasmids pGeneClip-shTCERG1-1, pGeneClip-shTCERG1-3, and pGeneClip-shTCERG1-4 (1:1:1). The control cells Jurkat shTCERG1-C1 were Jurkat stably transfected with the control vector pGeneClip-shTCERG1-C1. All three cell lines were grown in supplemented RPMI 1640 medium with 5 μg/ml puromycin (Invitrogen, Barcelona, Spain) at 37°C.

HEK293T cells were grown in DMEM (Dulbecco’s modified Eagle Medium*,* Gibco) with 10% fetal bovine serum (Gibco) supplemented with penicillin/streptomycin (100U and 0.1 mg/ml, respectively) and 4 mM L-glutamine (Gibco).

The Flp-In T-Rex-293 cell line is a tetracycline-inducible mammalian expression system (Invitrogen). Cells were maintained in high glucose DMEM with 10% fetal bovine serum supplemented with penicillin/streptomycin, 4 mM L-glutamine, and 15 μg/ml blasticidin (Invitrogen) to select for cells expressing the Tet repressor plasmid. To create inducible shRNA expressing cell lines, hairpin sequences targeting either GFP (control) or TCERG1 were cloned into pcDNA5/FRT/TO plasmid (Invitrogen) which contains a CMV promoter with two tandem repeats of the Tet operator. These constructs were transfected in the Flp-In T-Rex-293cells along with pOG44 which expresses the Flp recombinase. Stably transfected cells were selected with 200 μg/mL hygromycin and 15 μg/mL blasticidin. 5 μg/mL tetracycline (Sigma) was used for the induction of shRNA expression.

### Reagents and antibodies

IL-2 was used at 300 U/ml (Chiron, Emeryville, CA). Propidium Iiodide and 4′,6-diamidino-2-phenylindole (Dapi) were obtained from Sigma-Aldrich. Specific antibodies against TCERG1 have previously been described [51]. Monoclonal antibody against HIV-1 Tat (aa 2-9) was obtained from Advanced Biotechnologies Inc. (Columbia, MD). Specific antibodies against the β-isoform of actin were obtained from Sigma-Aldrich. Antibodies against CDK9 (C-20, sc-484), and RNAPII (N20, sc-899), TBP (N12, sc-204), CDK2 (M2, sc-163), and nucleolin (C23, sc-13057) were obtained from Santa Cruz Biotechnology. Anti-S5 and anti-S2 antibodies were obtained from Abcam. Secondary antibodies conjugated to horseradish peroxidase (HRP) were purchased from GE Healthcare Spain (Madrid, Spain). Secondary antibodies conjugated to Alexa-488 and Alexa-546 were purchased from Molecular Probes (Eugene, OR).

### Transfection assays

Stable transfection of Jurkat cells with pGeneClip-shTCERG1-C1 or pGeneClip-shTCERG1-3 and pGeneClip-shTCERG1-4 and/or pGeneClip-shTCERG1-1 vectors was performed by electroporation with an Easyjet Plus Electroporator (Equibio, Middlesex, UK). In brief, 5 × 10^6^ cells were collected in 250 μl of RPMI without supplements and mixed with 5 μg of DNA in an electroporation cuvette with a 2 mm electrode gap (Equibio). The cells were transfected by two pulses at 280 V, 150 μF, and 330 Ω. After transfection, the cells were incubated in supplemented RPMI for 18 h at 37°C. Puromycin was then added to the culture medium at 0.5 μg/ml to select stable shRNA expressing cells. For transient transfections, approximately 5-10 × 10^6^ cells were resuspended in 350 μl of RPMI without supplements and mixed with 5-10 μg of DNA in an electroporation cuvette with a 4 mm electrode gap (Equibio). Jurkat cells were transfected by one pulse at 280 V and 1500 μF and resting PBLs were transfected by one pulse at 320 V and 1500 μF. After transfection, the cells were incubated in supplemented RPMI for 18 h at 37°C. The HIV-1 p24 antigen was measured in cell culture supernatants by an enzyme-like immunoassay (InnotestTM HIV Ag mAb; Innogenetics, Barcelona, Spain). Luciferase and Renilla activities were assayed using the Luciferase Assay System (Promega), and transfection efficiency was monitored by flow cytometry to measure the quantity of cells expressing EYFP. Relative luciferase and Renilla units (RLUs) were measured in supernatants with a Sirius luminometer (Berthold Detection Systems, Oak Ridge, TN) after addition of the appropriate substrate. RLUs were normalized by measuring both β-Galactosidase activity and total protein concentration using the Bradford method [81].

For the RNA interference (RNAi) knockdown experiments, HEK293T cells were grown in 60-mm plates (Falcon) to 70-80% confluence and transfected using Lipofectamine 2000 reagent (Invitrogen) according to the manufacturers’ protocol with 60 nM of either one of the following small interfering RNA (siRNA) duplexes: siEGFP, 5′-CUACAACAGCCACAACGE-3′, or siTCERG1, 5′-GGAGUUGCACAAGAUAGUU-3′ [53]. Cells that did not receive siRNA were also used as a control. After 48 h, the plasmids pNL4-3-ΔRT and pXGH5 were co-transfected using Lipofectamine 2000. Cells were harvested 24 h later. Under these conditions, we consistently achieved at least ~80% knockdown of TCERG1.

### RNA extraction and RT-PCR assays

Total RNA was isolated using the RNeasy Mini kit (Qiagen) following manufacturer’s instructions. Specific primers for amplifying a 431 bp fragment of the 3′-end of the *tcerg1* gene were as follows: TCERG1-s, 5′-AAGGCCCGTTCAGAACAAACA-3′; and TCERG1-as, 5′-CGTCCTGAAGTCAGCTTTGGCT-3′. Primers for amplifying the human β-actin gene were as follows: β-actin-1 s, 5′-TCACCCACACTGTGCCCATCTA-3′, and β-actin-1as, 5′-AGTTGAAGGTAGTTTCGTGGAT-3′, yielding a fragment of 360 bp. The RT-PCR assay was performed in a C1000 Thermal Cycler (Bio-Rad Laboratories, Madrid, Spain) using the Access RT-PCR System (Promega). Briefly, the reaction mix contained 5 μl of 5x buffer, 2 mM MgCl_2_, 0.1 mM dNTPs, 0.4 μM of each primer pair, and 0.5 μl of each AMV Reverse Transcriptase and Tfl DNA Polymerase in a final volume of 25 μl. A total of 3 μg of RNA was added, and the thermal cycling conditions were as follows: an initial cycle of 48°C for 45 min and 95°C for 10 min; 29 cycles of 95°C for 30 sec, 51°C for 1 min, 68°C for 45 sec; and a final extension cycle of 68°C for 10 min. Amplified products were analyzed by electrophoresis on a 3% SeaKem agarose gel containing 5 μg/ml of ethidium bromide in 0.5x Tris-acetate buffer. The specificity of the amplified products was assessed by sequencing using an ABI Prism 3700 DNA Analyzer (Applied Biosystems, Foster City, CA).

For the analysis of the HIV genome elongation products (Figure 2B), the Jurkat-shTGERC1-(3-4), Jurkat-shTGERC1-(1-3-4) and Jurkat-shTGERC1-C1 cells were transfected with an infectious HIV-1 pNL4-3-wt clone and cultured for 48 h. Total RNA was isolated using the RNeasy Mini kit (Qiagen) following the manufacturer’s instructions, and 5 μg of RNA was used to synthesize cDNA with the GoScript Reverse Transcription System (Promega). Transcript elongation products were analyzed using SYBR Green PCR Master Mix in an ABI-Applied Biosystems 7500 Real Time Thermal Cycler (Applied Biosystems). The reaction mixture contained 10 μl of the PCR Master Mix and 0.4 μM of each primer pair in a final volume of 20 μl. One microliter of cDNA was added and amplified using the following conditions: an initial 10 min denaturation step at 95°C and 36 cycles consisting of 95°C for 15 sec, 60°C for 1 min, followed by a continuous melting curve stage. The following primers were used to amplify an early elongation product, yielding a fragment of 303 bp: R/U5 (sense), 5′-GGCTAACTAGGGAACCCACTGCTT-3′ (496-519) and gag (antisense), 5′-CTCGCACCCATCTCTCTCCTTCTA-3′ (777-799). To amplify a late elongation product, at the beginning of the *nef* gene, primers P3 (sense), 5′-TTGCTCAATGCCACAGCCAT-3′ (8654-8673) and P4 (antisense), 5′-TTTGACCACTTGCCACCCAT-3′ (8787-8806) [82] were used and a fragment of 152 bp was produced. Amplification of the gene encoding β-actin was used as an internal control for data normalization using the following primers: β-actin-2 s, 5′-AGGCCCAGAGCAAGAGAGGCA-3′; β-actin-2as, 5′-CGCAGCTCATTGTAGAAGGTGTGGT-3′, yielding a fragment of 114 bp. All amplified products were analyzed by electrophoresis on agarose gels and the specificity of amplified products was assessed by sequencing. Densitometry analysis was performed using Quantity One software (Bio-Rad). Gel bands were quantified and background noise was subtracted from the images. The relative ratio of optical density units was calculated relative to the gel band corresponding to the internal control for each lane and each type of RNA sample.

For the experiment shown in Figure 2C, total RNA was extracted from cells grown in 60-mm plates (Falcon) as previously described [53]. Approximately 3 μg of digested RNA was reverse transcribed using random hexamers. The quantification of the transcripts derived from the pNL4-3-ΔRT plasmid was performed by real-time PCR using the PerfeCTa SYBR Green SuperMix for iQ (Quanta Biosciences) and the iCycler thermal cycler station (Bio-Rad) with the following primers: ENV-F, 5′-TGGAAAACTCATTTGCACCA-3′ (8011-8027) and ENV-R, 5′-TTCTCTGTCCCACTCCATCC-3′ (8097-8116). Glyceraldehyde-3-phosphate dehydrogenase (GAPDH) was used as an internal control and was amplified using the following primers: GAPDHfwd, 5′-ATGGGGGAAGGTGAAGGTCG-3′ and GAPDHrev, 5′-GGGTCATTGATGGCAACAATATC-3′. pXGH5 was used as a transfection control and its products were amplified with the primers hGH-fwd, 5′-CAACAGAAATCCAACCTAGAGCTGCT-3′ and hGH-rev, 5′-TCTTCCAGCCTCCCATCAGCGTTTGG-3′.

### Purification of PICs and *in vitro* transcription/elongation assay

The purification of PICs and the *in vitro* transcription/elongation assay were performed as previously described [35].

### Immunoblotting assays

Protein extracts were obtained as previously described [83]. Protein concentration was determined by the Bradford method. Forty micrograms of protein extracts were fractionated by sodium dodecyl sulfate-polyacrylamide gel electrophoresis (SDS-PAGE) and transferred onto Hybond-ECL nitrocellulose paper (GE Healthcare). After blocking and incubation with primary and secondary antibodies, proteins were detected with SuperSignal West Pico/Femto Chemiluminescent Substrate (Pierce, Rockford, IL). Images were acquired using a Bio-Rad Gel Doc 2000 (Bio-Rad). Densitometry was performed as described above. For the experiment shown in Figure 4A, we used protocols that have previously been described [53].

### Immunofluorescence assays

For immunofluorescence assays, cells were immobilized in PolyPrep slides (Sigma-Aldrich) for 15 minutes and then fixed with 2% paraformaldehyde (PFA)-0.025% glutaraldehyde in PBS for 10 minutes at room temperature. After washing twice with 0.1% glycine/PBS, cells were permeabilized with 0.1% Triton X-100/PBS for 10 minutes. Cells were then treated with 1 mg/ml NaBH4 for 10 minutes. Incubation for 1 hour at room temperature with each primary and secondary antibodies and subsequent washes were performed with PBS1x-2% BSA-0.05% saponine buffer. Coverslips were immobilized with 70% glycerol/PBS. Images were obtained with a Radiance 2100 confocal microscope (BioRad, Hercules, CA), Leica TCS-SP confocal microscope (Leica Microsystems, Wetzlar, Germany) or a Leica DMI 4000B Inverted Microscope (Leica Microsystems).

### Chromatin immunoprecipitation assay

Control and TCERG1 knockdown T-Rex-HEK293 cells were seeded in 100-mm diameter plates at 70-80% confluence and transfected with 6 μg of pNL4-3∆RT plasmid. For overexpression experiments, T-Rex-HEK293 cells were additionally transfected with the pEFBOST7 empty vector or pEFBOST7-TCERG1 expression vector using the calcium phosphate precipitation method. After 48 h, the cells were fixed with 1% formaldehyde and incubated at room temperature for 10 min. The cross-link was arrested by adding glycine (0.125 M) for an additional 5 min at room temperature. Subsequently, the cells were pelleted, washed 3 times with phosphate-buffered saline (PBS), and lysed in SDS lysis buffer (1% SDS, 10 mM EDTA, 50 mM Tris-HCl [pH 8.1], protease inhibitor mixture [Complete; Roche], and 1 mM phenylmethylsulfonyl fluoride [PMSF]) for 10 min on ice. The lysates were sonicated 8 times for 15 s on ice and centrifuged at maximum speed. The sheared chromatin was diluted by the addition of 10 volumes of ChIP buffer (0.01% SDS, 1.1% Triton X-100, 1.2 mM EDTA, 16.7 mM Tris-HCl [pH 8.1], 167 mM NaCl, protease inhibitor mixture, and 1 mM PMSF) and pre-cleared with a salmon sperm DNA/protein A-agarose fast-flow slurry (Millipore) for 2 h. The beads were removed by centrifugation. 5% of the pre-cleared chromatin was removed to serve as the pre-immunoprecipitation (pre-IP; input) control, and the remaining pre-cleared chromatin was incubated overnight with 5 μg of the specific antibodies or nonspecific rabbit IgG. The chromatin-antibody complexes were collected by incubation with salmon sperm DNA/protein-A agarose (50% slurry) and centrifugation. Beads were washed in low or high salt conditions using buffer (0.1% SDS, 1% Triton X-100, 2 mM EDTA, 20 mM Tris-HCl [pH 8.1], and 150 mM NaCl) containing 20 mM and 500 mM NaCl, respectively. The beads were then washed once with LiCl buffer (0.25 M LiCl, 1% NP-40, 1% Na-deoxycholate, 1 mM EDTA, and 10 mM Tris-HCl [pH 8.0]) followed by two washes with Tris-EDTA buffer. The antibody-chromatin complexes were eluted from the beads by incubation with elution buffer (0.1% SDS, 0.1 M NaHCO_3_). A final concentration of 0.2 M NaCl was added to eluates, and the samples were incubated at 65°C for 4-6 h. The samples were treated with RNase A and proteinase K, and the DNA was purified using phenol-chloroform extraction. The pre-IP input sample was purified in a similar manner. The DNA obtained was amplified by quantitative PCR (qPCR) using PerfeCTa SYBR Green SuperMix for iQ (Quanta Biosciences). The following primers were used: 5′-TAGTGTGTGCCCGTCTGTTG-3 (554-573) and 5′-CGCTTTCAAGTCCCTGTTCG-3′ (643-662) for the 5′ LTR; 5′-AAAGGGCTGTTGGAAATGTG-3′ (2019-2038) and 5′-GGCTCTGGTCTGCTCTGAAG-3′ (2131-2153) for the *gag* gene; 5′-GTTTGGAAAGGACCAGCAAA-3′ (4929-4948) and 5′-CACAATCATCACCTGCCATC-3′ (5051-5070) for the *vif* gene; 5′-TGGAAAACTCATTTGCACCA-3′ (8011-8027) and 5′-TTCTCTGTCCCACTCCATCC-3′ (8097-8116) for the *env* gene; 5′-GGTGGGTTTTCCAGTCACAC-3′ (8981-9000) and 5′-GGGAGTGAATTAGCCCTTCC-3′ (9077-9096) for the 3′ LTR. Dilutions of the input were used to normalize the obtained values. The results represent the average of four independent experiments.

### RNAPII processivity assay

The rates of RNAPII transcription were measured as previously described [53,54] with minor modifications. Briefly, control and TCERG1 knockdown T-Rex-HEK293 cells were grown to approximately 70 to 80% confluence and transfected with 1 μg of pNL4-3-ΔRT and pXGH5 plasmids using Lipofectamine 2000 (Invitrogen) according to the manufacturers’ protocol. The next day, the cells were treated with 100 μM 5,6-Dichlorobenzimidazole 1-β–D-ribofuranoside (DRB) (Sigma) for 3 h. The cells were washed with PBS to remove the DRB and incubated in fresh medium for various periods of time. Total RNA was then isolated using TRIzol reagent. The cDNA was amplified by qPCR using the PerfeCTa SYBR Green SuperMix for iQ (Quanta Biosciences) with primers ENV-F and ENV-R. GAPDH was used as a reference gene control and amplified with GAPDHfwd and GAPDHrev. Amplification of pXGH5 with the primers hGH-fwd and hGH-rev was used as transfection control. The *CT* was calculated as follows: (*E* pNL4-3-ΔRT) ^Δ*CT* pNL4-3-ΔRT^/(*E* GAPDH) ^Δ*CT* GAPDH^, where *E* is the PCR efficiency and Δ*C*_*T*_ = *C*_*T*_ for the control – *C*_*T*_ for DRB-treated cultures.

### Statistical analysis

Statistical analysis was performed using Graph Pad Prism 5.0 (Graph Pad Software Inc., San Diego, CA) with one-way analysis of variance (ANOVA) followed by Bonferroni post-test analysis to describe the significant differences between groups. *p*-values (*p*) < 0.05 were considered statistically significant in all comparisons and are represented as *, ** or *** for *p* < 0.05, *p* < 0.01 or *p* < 0.001, respectively.

## Abbreviations

TCERG1: Transcription elongation regulator factor 1; RNAPII: RNA polymerase II; CTD: Carboxyl-terminal domain; LTR: Long terminal repeat; Ser: Serine; PICs: Pre-initiation complexes.

## Competing interests

The authors declare that they have no competing interests.

## Authors’ contributions

MC, CHM, JA, and CS conceived the study. MC, MM, IM, MRLH, and EM performed the experiments. CLS and MAGB generated the Flp-In T-Rex-293 cell lines used in the analyses. All authors contribute in writing the paper. All authors have read and approved the submission of the manuscript.

## Supplementary Material

Additional file 1: Figure S1 Effect of silencing and overexpression of TCERG1 on the expression and phosphorylation of RNAPII. Expression of RNAPII and its phosphorylation on ser2 was analyzed by Western blot in Jurkat cells with stable TCERG1 mRNA interference (Jurkat shTCERG1-(3-4) and Jurkat shTCERG1-(1-3-4)), in comparison with control cells Jurkat shTCERG1-C1 (A) and in Jurkat cells with transient overexpression of TCERG1, in comparison with cells transfected with an empty pcDNA3 vector (B). β-actin was used as loading control. Click here for file

Additional file 2: Figure S2 Overexpression of CDK9 did not rescue the LTR-dependent transcription in the absence of TCERG1. Jurkat cells with stable TCERG1 mRNA interference (Jurkat shTCERG1-(1-3-4)) were co-transfected with LTR-LUC and a CDK9 expression vector (wild-type (wt) or mutant (dn)) (right panel), in comparison with control cells Jurkat shTCERG1-C1 (left panel). Luciferase activity was measured and represented as RLUs; the data are expressed relative to the cells transfected with pcDNA3. The CDK9 overexpression was determined by Western blot. In all cases, the data from three different experiments is represented in the histograms (mean ± SD). Statistical analyses were performed and are shown as *, p < 0.05; **, p < 0.01; ***, p < 0.001. Click here for file
