# Supplementary figures and images for: Transcription elongation regulator 1 (TCERG1) regulates competent RNA polymerase II-mediated elongation of HIV-1 transcription and facilitates efficient viral replication
Source: Retrovirology. 2013 Oct 28;10:124. doi: 10.1186/1742-4690-10-124 (PMC3874760; doi:10.1186/1742-4690-10-124)

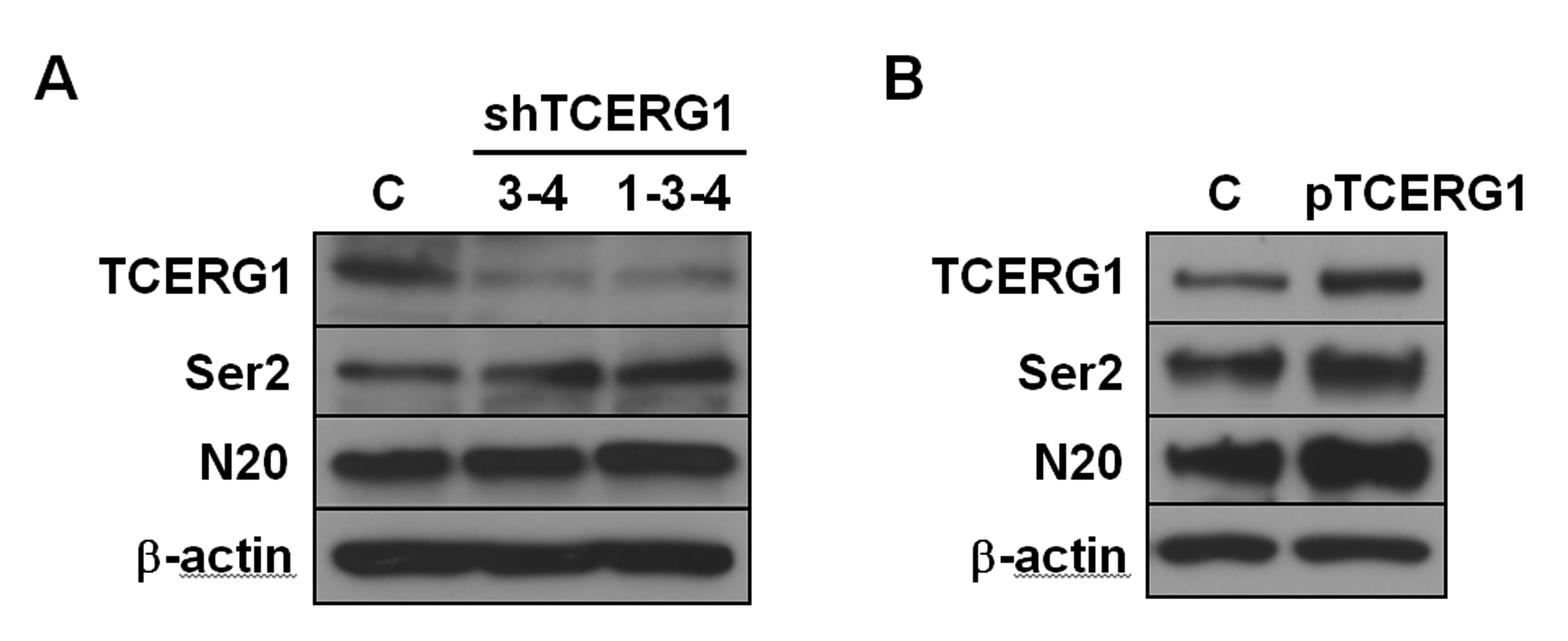

Supplement: Additional file 1: Figure S1 — Effect of silencing and overexpression of TCERG1 on the expression and phosphorylation of RNAPII. Expression of RNAPII and its phosphorylation on ser2 was analyzed by Western blot in Jurkat cells with stable TCERG1 mRNA interference (Jurkat shTCERG1-(3-4) and Jurkat shTCERG1-(1-3-4)), in comparison with control cells Jurkat shTCERG1-C1 (A) and in Jurkat cells with transient overexpression of TCERG1, in comparison with cells transfected with an empty pcDNA3 vector (B). β-actin was used as loading control. [file 1742-4690-10-124-S1.tiff]

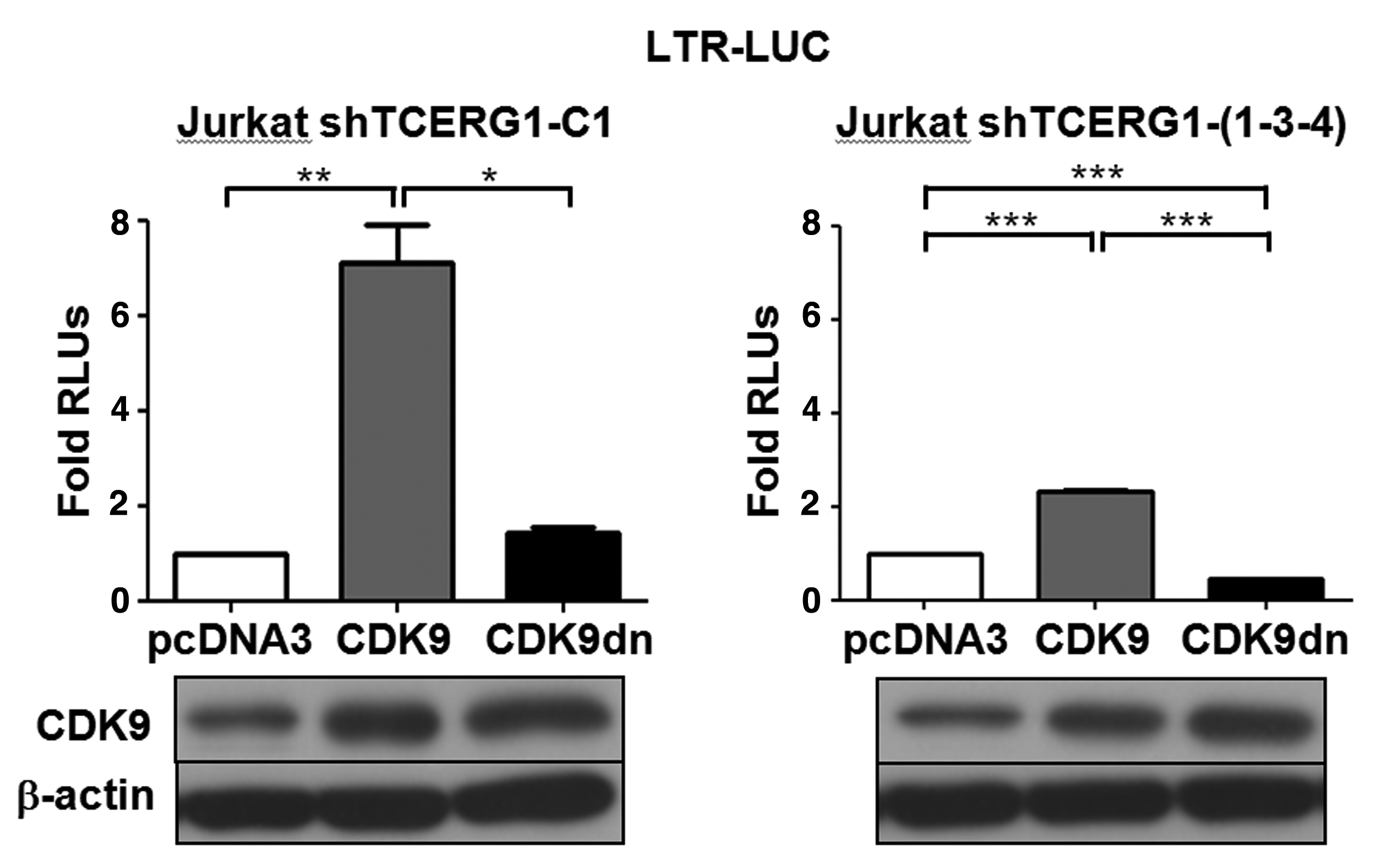

Supplement: Additional file 2: Figure S2 — Overexpression of CDK9 did not rescue the LTR-dependent transcription in the absence of TCERG1. Jurkat cells with stable TCERG1 mRNA interference (Jurkat shTCERG1-(1-3-4)) were co-transfected with LTR-LUC and a CDK9 expression vector (wild-type (wt) or mutant (dn)) (right panel), in comparison with control cells Jurkat shTCERG1-C1 (left panel). Luciferase activity was measured and represented as RLUs; the data are expressed relative to the cells transfected with pcDNA3. The CDK9 overexpression was determined by Western blot. In all cases, the data from three different experiments is represented in the histograms (mean ± SD). Statistical analyses were performed and are shown as *, p < 0.05; **, p < 0.01; ***, p < 0.001. [file 1742-4690-10-124-S2.tiff]
